# The effects of media and peers on negative body image among Chinese college students: a chained indirect influence model of appearance comparison and internalization of the thin ideal

**DOI:** 10.1186/s40337-022-00575-0

**Published:** 2022-04-12

**Authors:** Jianting Shen, Jinjun Chen, Xiwen Tang, Shangfei Bao

**Affiliations:** Department of Psychology, Hunan University of Science and Technology, Xiangtan, 411201 China

**Keywords:** Peer, Media, Appearance comparison, Internalization of the thin ideal, Body image

## Abstract

**Background:**

Negative body image is a common psychological phenomenon among Chinese college students meriting investigation. Peers and the media are important factors that negatively influence body image. This study explored the mechanisms of media and peers promoting negative body image among Chinese college students.

**Method:**

Data from 407 college students (173 men and 234 women) were collected using the Revised Social and Cultural Attitude Questionnaire of Appearance, Appearance Comparison Scale, Peer Impact Scale, and Negative Body Image Scale.

**Results:**

Correlational analysis results demonstrated that media attention was not significantly correlated with negative body image. All other variables were significantly positively correlated. Path analysis results indicated that the direct effect of media attention on negative body image was not significant, while the direct effect of peer impact was significant. In addition, appearance comparison and internalization of the thin ideal acted as a chained indirect effect between negative body image, media attention, and peer impact.

**Conclusions:**

The research revealed that focusing on perfect bodies displayed in the media did not produce a negative body image. However, focusing on the media and peer conversations regarding the body caused the participants to compare appearances and internalize ideal body shape standards, leading to negative self-evaluations.

## Plain English Summary

Adolescents’ peers and media have far-reaching influence. This study explored their impact on the body image of Chinese college students. A questionnaire survey was distributed to 461 Chinese college students. The results showed that peers directly affected body image, while media attention did not. Media attention and peer impact further increased students’ negative body image evaluations through the indirect effects of appearance comparison and internalization of the thin ideal.

## Introduction

**Fig. 1:**
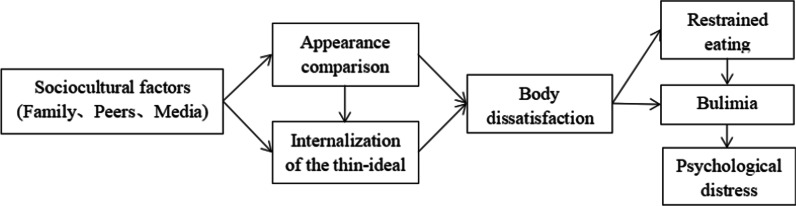
The tripartite influence model

Body image is an individual’s attitude regarding their own body size, shape, and aesthetic feeling [1]. An individual with a negative body image dislikes and denigrates their own body’s size and other dimensions. For example, a survey of students from grade one of junior school to the fourth year of university in China indicated a high prevalence of dissatisfaction with physical appearance [2]. The tripartite influence model is a sociocultural theory proposed to explain various factors’ effects on body image [3]. This model hypothesizes that sociocultural factors, particularly family, peers, and media, affect satisfaction with physical appearance. In addition, appearance comparison and internalization of the thin ideal are mediators between social and cultural factors and physical appearance [3] (Fig. 1).

The Tripartite Influence Model was developed in the Western cultural background, and whether it is suitable for the Chinese cultural background requires further verification. Collectivism is embedded in the social orientation of Chinese people [4]. In current Chinese and Western societies, adolescents and college students especially have low body satisfaction [5, 6]. A survey of college students' body satisfaction by Guo and his colleagues shows that most college students are not very satisfied with their body shape. They are dissatisfied to some degree with certain parts of the body. On the one hand, this stems from the mentality of comparing with and striving to attain a “perfect” appearance. On the other hand, the aesthetic orientation of modern people also affects the satisfaction of college students with various parts of the body [7].

The social orientation of people in China can be divided into four categories: relationship, authority, family (group), and others. Social orientation refers to a set of adaptive lifestyles through which individuals integrate into or cooperate with their social environment [8]. An others orientation refers to a tendency to experience the psychological and behavioral influence of others. This tendency includes being particularly sensitive to opinions, standards, praise, and criticism, hoping to make a good impression, and striving to conform with others [8]. Chinese people tend to focus on other people’s thoughts and evaluations and readily feel interpersonal pressure [9], reflecting a desire to avoid differences and protect oneself. Unlike Western cultures, Chinese culture appears more concerned with image, including appearance, which may lead to the pursuit of social norms or standards set by others, resulting in negative self-evaluation. Therefore, mass media and peers easily influence the body image of Chinese college students in their collectivist culture. This potentially negative influence of mass media and peers on body image in Chinese society should be investigated [10].

Beginning junior high school, most students in China live on campus independent from their families; some have lived independently since primary school. As life centers mainly on campus, they spend the majority of their time with peers. Thus, the influence of peers on college students gradually increases [11]. Moreover, parental attitudes have little impact on an individual’s body image [12]. For these reasons, this study explored only the influence of mass media and peers on negative body image. In addition, it attempts to verify further the applicability of the tripartite model in collectivist cultures.

### The effects of media and peers on negative body image

Mass media include carriers, tools, and technical means, such as magazines, newspapers, periodicals, books, television, movies, radio, audio-visual products, and the Internet [13]. It also includes organizations engaged in information collection, processing, and dissemination, such as newspapers, websites, major TV stations, and other media organizations [13, 14]. This study used a broad definition of mass media, including the former and latter categories. Electronic media, now more pervasive than older print forms, has an especially powerful impact on youth Mass media is an important factor that may negatively affect body image [15], especially that of women [16]. Myers and Biocca [17] found that watching advertisements or programs related to slimming for only one minute could change a woman’s view of her own body. Due to perceived media pressure, some women think that slim figures significantly influence personal attraction and relate to career success, good interpersonal relationships, and achievement of ideals [18]. According to the self-objectification theory [19], the media constantly portrays slim and slender female images, causing women to look at their physical self from a third-person perspective and focus on observable physical attributes rather than evaluating themselves in terms of their abilities. Women may adopt an attitude of scrutinizing their bodies intensively, leading to negative psychological or subjective experiences. Furthermore, adults dissatisfied with their health have a cognitive bias toward health-related information [20–22], including attention, memory, and explanation biases. Compared with people with low levels of body dissatisfaction, people with high levels of body dissatisfaction pay more attention to body parts they find unattractive. They also pay more attention to body parts that are considered most attractive when looking at others [21]. Restrained eaters showed better recognition of body-related images that they had previously seen during the visual search task. [20]. Frequent exposure to unrealistic standards of the ideal body shape conveyed by the media may affect how individuals process body-related information, making it easier for them to form negative body images [21].

College students’ social circles are mainly campus-based. Their peers are the main social objects. Communication with peers affects focus on appearance, formation of ideal body shape standards, and development of body images [23]. In this study, peer impact refers to the influence of peers’ conversations, evaluation, and ridicule on body appearance. Conversations regarding obesity among women, often occurring in women’s friendship groups and focus on body size, weight, and eating habits, have an adverse impact on body image cognition [24]. In addition, studies have shown that peer ridicule is an essential predictor of dissatisfaction with physical appearance [25].

### A chained indirect influence model of appearance comparison and internalization of the thin ideal

Mass media is a powerful vector for spreading sociocultural standards and expectations, such as ideal size, weight, and fashion [26]. With its rapid development, the number of college students who access pictures and articles promoting an “ideal body image” is rapidly increasing. According to Social Comparison Theory, comparative social motivation toward physical attention is the main factor promoting dissatisfaction with personal appearance [27, 28]. If an individual looks at ideal body images, especially when they consider their physique inferior, they experience upward social comparison and self-evaluation motivation. The aesthetic standard of thinness as beauty has become common for most people, leading to a growing number of people becoming dissatisfied with their body shape, resulting in negative body image. “Be thin or die” has become the motto of many young women today [29]. Internalization of the thin ideal is the process of adopting a socially perfect body image as a personal goal and standard [3]. College students are influenced by the definition of the “perfect figure” by mass media for a long time and may constantly internalize the social perfect appearance standard [30]. Research has shown that female viewers’ dissatisfaction with physical appearance increases immediately after watching music TV programs featuring slim female images [31]. Other studies have shown that the higher the female internalization level of the ideal thin female model image in fashion magazines, the stronger the negative body image of female college students [30]. In addition, research has shown that repeated exposure to visual images of ideal thin models leads to higher internalization levels. This internalization then leads to dissatisfaction with physical appearance [32, 33]. Such internalization has indirect effects on sociocultural influences and negative body image of women and their subsequent behaviors. According to the tripartite influence model mentioned above, appearance comparison, which involves comparing oneself with images of ideal thinness spread in the media, positively predicts the internalization of the thin ideal [3]. In the comparison process, the individual constantly accepts the ideal body shape promoted by the mass media and gradually internalizes the standard of perfect thinness. Therefore, college students’ exposure to the thinness ideal disseminated by mass media may lead to appearance comparison and internalizing social standards, eventually producing negative body images.

Compared with children, teenagers focus more on their appearance and image and other people’s evaluation of themselves and are sensitive to their external “shortcomings” or “imperfections.” Peers’ ridicule regarding appearance further deepens teenagers’ awareness of their “imperfections” [34]. Studies have shown that female college students’ involvement in obesity reviews can positively predict negative physical self-image [35]. According to Social Comparison Theory, hearing negative comments or talking about one’s figure causes upward social comparison and self-evaluation motivation [36]. The negative comments on peers’ figures stem from the fact that peer groups have “perfect figure” standards. Poor evaluation forces them to compare themselves with the “perfect figure,” thus producing more negative body images. Peer conversations about the body constantly strengthen these social standards and change an individual’s view of their body. According to a previously mentioned study [9], the viewpoint that “thinness is beauty” is frequently applied in evaluating body size in various interpersonal relationships. Thus, the social pressure generated has a significant impact on individual body images. A longitudinal study of adolescent boys and girls demonstrated that internalization mediates the relationship between peer appearance conversations and dissatisfaction with physical appearance [37]. Currently, many college students upload their photos to social platforms. They receive comments on their appearance from peers and others, including negative ones. They read intentionally or unintentionally harmful reactions, such as “You look a little fatter in that dress.” According to Social Comparison Theory and the sociocultural model, this feedback forces comparisons with “better” body shapes and internalization of the “better” body standard, which is difficult to achieve. This process induces a negative body image.

Therefore, this study explored the relationship between media attention, peers, and negative body images and the chained indirect effect of college students’ appearance comparisons and internalizations of the thin ideal. In addition, this study examined the applicability of the tripartite influence model in China.

### Present study

This study had two objectives. First, to explore the relationship between media attention, peer influence, and negative body image among college students. Second, to determine whether the relationships between media attention, peer influence, and negative body image are indirectly affected by appearance comparison and internalization of the thin ideal.

#### **Hypothesis 1**

Media attention among college students positively predicts negative body image.

#### **Hypothesis 2**

Peer influence among college students positively predicts negative body image.

#### **Hypothesis 3**

Comparison and internalization of the thin ideal relate to a chained indirect influence of media attention on negative body image.

#### **Hypothesis 4**

Comparison and internalization of the thin ideal relate to a chained indirect influence of peer influence on negative body image.

## Methods

### Participants

This study used convenience sampling to distribute 461 questionnaires at a university in Hunan, China, and received 407 valid questionnaire responses, with a response rate of 88.29%. The participants included 173 men (42.5%) and 234 women (57.5%). Of the participants, 125 were freshmen (30.7%), 97 were sophomores (23.8%), 79 were juniors (19.4%), and 106 were seniors (26.0%). The average age was 20.23 years, and the standard deviation was 1.61.

### Measures

#### Media attention and internalization of the thin ideal

Media attention and internalization of the thin ideal were measured using the Sociocultural Attitudes Towards Appearance Scale-3 [38]. The scale contains 15 items in two subscales: Media Attention Subscale (six questions, such as, “I pay attention to the information related to personal attractiveness in magazines”) and Internalization Subscale (nine questions, such as, “I wish my body looked like the people in the movies”). Items 2, 5, 6, 10, and 11 were reverse-scored, and the five-point scoring method was adopted (1 = *completely disagree*, 5 = *completely agree*). Higher scores indicated higher internalization and media attention. Kehlenbach’s coefficient of the scale was 0.89.

#### Peer impact

Peer impact was measured using the Peer Impact Scale in the Sociocultural Attitudes Towards Appearance Scale-3 [39]. The scale comprises 13 items, translated and revised into 12 items, such as, “Have your friends or classmates ever commented about or laughed at your appearance?” Answers were rated on a five-point scale (1 = *never*, 5 = *always*). Higher scores indicated a greater negative influence from peers. Kehlenbach’s coefficient of the scale was 0.89.

#### Appearance comparison

Appearance comparison was measured using the Chinese version of the Appearance Comparison Scale [6]. The scale included five items, such as, “At parties or other social activities, I compare my appearance with that of others,” with answers rated on a five-point scale (1 = *never*, 5 = *always*). Higher scores indicated higher social comparative tendency. Kehlenbach’s coefficient of the scale was 0.77.

#### Negative body image

Negative body image was measured using the Negative Body Image Scale [40], which included 27 items for four factors: overall (three items, such as, “I am proud of my body”), fatness (nine items), appearance (seven items), and shortness (eight items). Items 2, 3, 11, and 23 were reverse-scored. The answers were rated on a five-point scale (1 = *completely disagree*, 5 = *completely agree*). Higher scores indicated more intense dissatisfaction with body image. Kehlenbach’s coefficient of the scale was 0.84.

### Data collection and statistical analysis

The data was collected using a questionnaire survey. It was shared primarily in the school's QQ contact group in the form of an online questionnaire. All research participants were informed that the questionnaire survey was anonymous. The collected data would be used only for scientific research with strict confidentiality.

SPSS 24.0 and AMOS 26.0 were used to conduct correlation and path analyses.

## Results

### Preliminary analyses

Harman’s single-factor analysis was used to test the deviation of the common method [41]. The results demonstrated that 11 factors had eigenvalues greater than 1 without rotation, which explained 66.94% of the variation. In addition, the first factor explained 26.36% of the variation, which was less than the standard recommended by previous studies (30%). Therefore, no substantial common method deviations were observed in this study.

There was no gender difference in the negative body image of college students (*p* > 0.05). There was also no grade difference in the negative body image of college students (*p* > 0.05). Although media attention was not significantly related to negative body image, the other variables were significantly positively related (Table 1).

**Table 1:** Descriptive and correlations for variables

Variable	M ± SD	1	2	3	4	5
Media attention	3.21 ± 0.73	1				
Peer impact	3.04 ± 0.69	0.152**	1			
Appearance comparison	2.88 ± 0.84	0.168**	0.423**	1		
Internalization of the thin-ideal	2.89 ± 0.72	0.350**	0.328**	0.532**	1	
Negative body image	2.57 ± 0.62	0.093	0.377**	0.420**	0.479**	1

*p < .05; **p < .01; ***p < .0001

### Main analyses

Based on previous studies and the relationship analysis between variables, this study established a chained indirect effect model. It investigated the indirect predictive mechanism of appearance comparison and internalization of the thin ideal in the relationship between media attention, peer impact, and negative body image. The fit index of this model was χ^2^ = 4.138, χ^2^/df = 4.138, NFI = 0.99, CFI = 0.99, IFI = 0.99, RMSEA = 0.08, indicating that the model was well-adapted and acceptable.

**Fig. 2:**
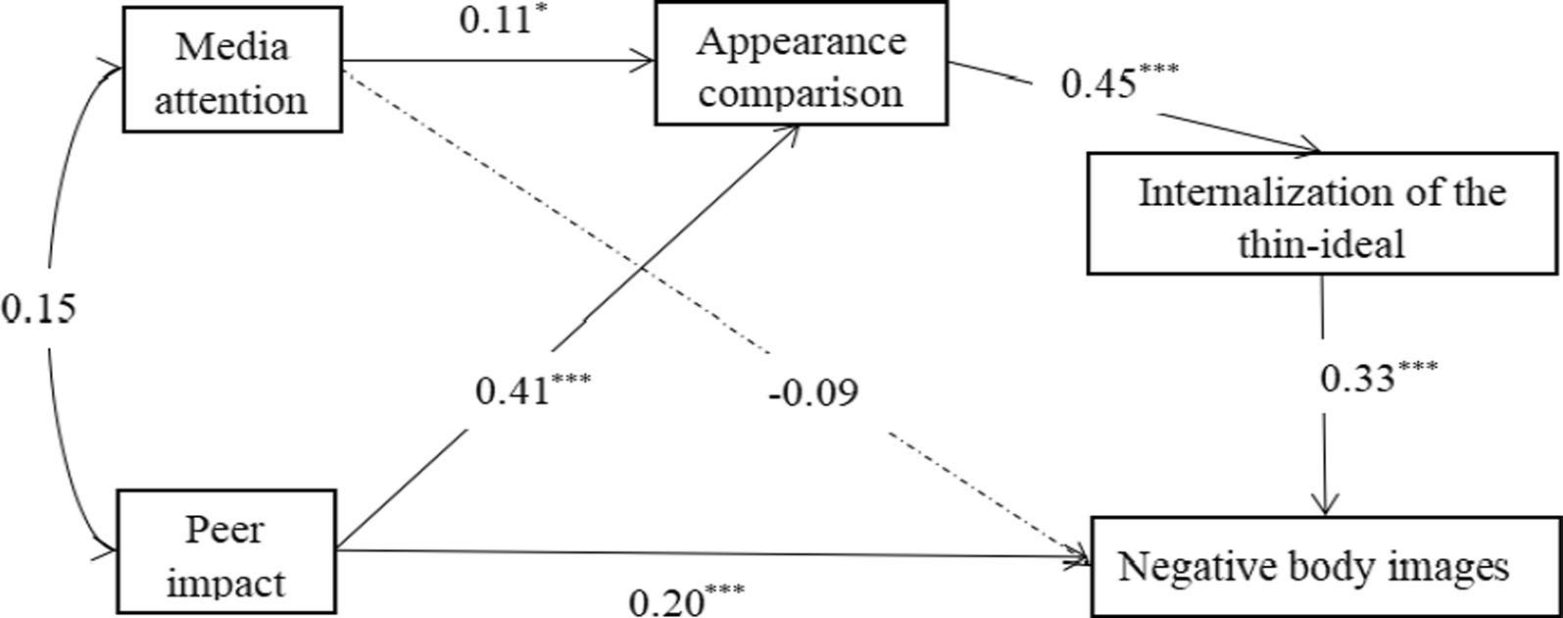
A chained indirect influence model of appearance comparison and internalization of the thin ideal. Note. Standardized regression weights were reported. The dotted lines depict non-significant paths. *p < 0.05; **p < 0.01; ***p < 0.001

The results revealed that the direct effect (Fig. 2) between media attention and negative body image was not significant. In contrast, the direct effect between peer impact and negative body image was significant. Furthermore, the indirect influence of appearance comparison and internalization of the thin ideal were significant, and the chained indirect effects of media attention on negative body image and peer impact on negative body image were significant. This result indicated that appearance comparison and internalization of the thin ideal had chained indirect effect of media attention and peer impact on negative body image.

The path coefficient (Fig. 2) showed that media attention was negatively correlated with negative body image and positively predicted appearance comparison (*β* = 0.11, *p* < 0.05). Peer impact positively predicted negative body image (*β* = 0.20, *p* < 0.001). Appearance comparison positively predicted internalization of the thin ideal (*β* = 0.45, *p* < 0.001). Internalization of the thin ideal positively predicted negative body image (*β* = 0.33, *p* < 0.001).

Based on the significant coefficient of the direct path, an indirect effect was tested. Furthermore, the bootstrap program of AMOS tested the significance of the indirect effect. The confidence intervals corresponding to each path did not contain 0 (Table 2), indicating that the indirect effects were significant. The participants’ appearance comparison and internalization of the thin ideal had indirect effects on media attention, peer impact, and negative body image.

**Table 2:** Bootstrap analysis of the significance of the indirect effect

Path	Estimate (S.E.)	95%CI	
		LL	UL
Media attention → Appearance comparison → Internalization of the thin ideal → Negative body image	0.015 (0.008)	0.002	0.035
Peer impact → Appearance comparison → Internalization of the thin ideal → Negative body image	0.059 (0.012)	0.039	0.090

LL, lower limit; UL, upper limit

## Discussion

This study investigated the effects of media attention and peers on negative body images and the indirect effect of appearance comparison and internalization of the thin ideal. The results demonstrated that peer impact significantly positively predicted negative body image. Furthermore, appearance comparison and internalization of the thin ideal had chained indirect effects of peer impact and media attention on negative body image.

Surprisingly, this study found no significant correlation between media attention and negative body image. Therefore, Hypothesis 1 was not supported, consistent with other research results. One study observed no inevitable relationship between an ideal thin image of media communication and low physical satisfaction [42]. Compared with a negative body image, the media had a more remarkable effect on spreading a positive body image [43]. This study indicated that focusing only on the information of a perfect figure in mass media did not necessarily produce negative body images, which might relate to its internal mechanism. Media attention does not directly affect negative body images; negative body image results from comparing oneself with and internalizing media standards. This study focused on revealing the indirectly predictive mechanism.

Furthermore, the results indicated that appearance comparison and internalization of the thin ideal had a chained indirect impact on media attention and negative body image. Media attention caused negative body evaluation through appearance comparison and internalization of the thin ideal supporting Hypothesis 3. Online media efficiently spread images of the ideal figure in modern society and market the social standard of “thinness is beauty.” As Chinese people live within a collectivist culture, they have more characteristics of social orientation, are more easily influenced by the network environment, and strive to match the social standards of the ideal figure. Thus, actively and frequently comparing one’s actual body image with the beautified pictures of others (i.e., making comparisons with someone slimmer) causes individuals to focus on their physical deficiencies, resulting in the harmful idea that others are better people. According to Upward Assimilation Theory [44], people tend to compare themselves with those with superior abilities and attributes than themselves, producing negative self-evaluation. Therefore, the participants’ focus on the perfect image in the media causes upward social comparison. People with stronger social inclinations are more likely to compare themselves with the notion of “ideal thinness” spread in the media [12]. In addition, according to the Tripartite Influence Model, appearance comparison predicts the internalization of the thin ideal. Specifically, the more information an individual receives from mass media about their ideal figure, the easier it is to compare and internalize this information upwards, and the higher the degree of internalization. Therefore, individuals are more likely to be dissatisfied with their bodies, in line with previous studies [45]. This result suggested that individuals who focus excessively on body information in the mass media are likely to have upward comparison motivation. They compare their bodies with those of models or stars. Also, individuals with a higher tendency to compare appearances are more likely to internalize ideal bodies. Internalization may occur because traditional Chinese people have a strong psychological inclination to avoid differences and seek common ground. They have a strong tendency toward social conformity [8]. Therefore, the participants likely wanted their bodies to be consistent with social standards. However, individuals are often dissatisfied with their bodies because they cannot achieve this standard. Focusing only on media information did not produce negative body images. However, the internal mechanism of appearance comparison and internalization produced negative body images.

In addition, the results revealed that peer impact positively predicted negative body images, supporting Hypothesis 2. “Social orientation is the way that Chinese people operate or adapt in their life circle” [46, p. 26]. Social orientation enables people to behave in accordance with social expectations, gain approval from others, and maintain interpersonal relationships. Under the influence of peers’ appearance comments, college students judge themselves according to the social appearance standards to obtain good interpersonal relationships and praise. Frequent conversations about obesity in women’s friendship groups were “self-derogatory” [47], and regular participation in the talk about obesity tends to “enhance the negative experience of women’s physical dissatisfaction” [48]. Research has demonstrated that more severe experiences of peer mockery result in higher dissatisfaction with physical appearance and more negative body image [49]. Tucker et al. [50] investigated how women with differing levels of self-esteem performed in three different body image description groups. They found that among the peer groups that belittle each other, women experienced the lowest satisfaction with their bodies. In addition, research has shown that women with negative body images have attentional biases toward negative appearance words. This tendency may be difficult to overcome [51]. Therefore, the results of this study indicated verbal interaction between peers regarding body size and weight aggravated individual dissatisfaction with body size.

Finally, appearance comparison and internalization of the thin ideal had an indirect prediction on peer influence and negative body image, supporting Hypothesis 4. This result indicated that participants who were the focus of comments or excessive evaluation by friends and classmates regarding their weight, weight loss, or appearance engaged in comparison among peers, thus internalizing others’ definition of a “good figure” and even negatively evaluating their own figures. Due to social orientation, Chinese people are sensitive to others’ opinions [9]. In line with this orientation, our study results demonstrated that peer opinions influenced male and female college students. They are concerned about their evaluation of their figure, especially if negative. Chinese people attach importance to their reputation and face. For traditional Chinese people, others are omnipresent listeners and audiences. Chinese people are not only keen social information searchers but also smart self-presenters. Collecting information about oneself and frequent self-monitoring constitute processes of comparison, internalization, and constant adjustments to self-presentation to achieve a good reputation [8]. Therefore, the participants collected appraisals of their figures from peers and classmates and conducted self-evaluations, producing mental comparisons with a hypothetical “good body.” Subsequently, they internalized the body standards of peers and society, affecting their confidence levels and causing negative emotions and poor body images.

## Strengths and limitations

This study made several contributions to the literature. First, it explored the effects of mass media and peer impact on negative body image and its underlying mechanism. This study further enriched Thompson’s sociocultural model by applying it to Chinese college students. This design will greatly enhance our understanding of the effects of mass media and peer impact on the body image of college students. Additionally, it will guide them to achieve positive physical and mental health. First, excessive discussion and evaluation of body weight among peers caused negative body images. Therefore, to reduce peer comments on each other’s figures, college students should be guided to evaluate themselves according to their internal characteristics such as ability and morality rather than appearance. Second, excessive comparison and internalization of appearance promotes negative body images. Therefore, we should guide students to form stable self-concepts that will be resistant to external standards, as appearance is not a standard for measuring a person’s value. Third, this study verified the applicability of Thompson’s sociocultural model to Chinese college students.

However, this study had some limitations. First, this was a cross-sectional study, and it was impossible to determine the causal relationships among variables. Future studies should apply experimental methods to detect cause-and-effect connection among variables. Second, the participants selected in this study were college students. Future research could include other age groups to explore the influence of mass media and peers on different age groups. Finally, this study considered only two mediators in Thompson’s sociocultural model. Whether other variables promote negative body image remains to be determined.

## Conclusions

This study confirmed the specific influence mechanism of media and peer factors on Chinese college students using a sociocultural model. The results demonstrated that peer influence has a direct, detrimental effect on the body images of college students. However, the negative impact of media attention on college students’ body images may not be direct. College students negatively evaluate their bodies through psychological activities, such as appearance comparison and idealization. In addition, media attention and peer impact further increased the negative evaluation of college students’ figures through the chained indirect effect of appearance comparison and internalization of the thin ideal.

## Data Availability

The datasets generated and analyzed during the current study are available from the corresponding author upon reasonable request.
